# C_1_-Pathways in *Methyloversatilis universalis* FAM5: Genome Wide Gene Expression and Mutagenesis Studies

**DOI:** 10.3390/microorganisms3020175

**Published:** 2015-04-09

**Authors:** Nathan M. Good, Andrew Lamb, David A. C. Beck, N. Cecilia Martinez-Gomez, Marina G. Kalyuzhnaya

**Affiliations:** 1 Department of Microbiology, University of Washington, Seattle, WA 98195-1700, USA; E-Mails: nategood@uw.edu (N.M.G.); andrewelamb@gmail.com (A.L.); 2 Department of Chemical Engineering, University of Washington, Seattle, WA 98195-7735, USA; E-Mails: dacb@uw.edu (D.A.C.B.); mart1754@msu.edu (N.C.M.G.); 3 eScience Institute, University of Washington, Seattle, WA 98195-1570, USA; 4 Biology Department, San Diego State University, North Life Science Room 401, San Diego, CA 92182-4614, USA

**Keywords:** rhodocyclaceae, *methyloversatilis*, C_1_-metabolism, *N*-methylglutamate pathway, formaldehyde activating enzyme homologues

## Abstract

*Methyloversatilis universalis* FAM5 utilizes single carbon compounds such as methanol or methylamine as a sole source of carbon and energy. Expression profiling reveals distinct sets of genes altered during growth on methylamine *vs* methanol. As expected, all genes for the *N*-methylglutamate pathway were induced during growth on methylamine. Among other functions responding to the aminated source of C_1_-carbon, are a heme-containing amine dehydrogenase (Qhp), a distant homologue of formaldehyde activating enzyme (Fae3), molybdenum-containing formate dehydrogenase, ferredoxin reductase, a set of homologues to urea/ammonium transporters and amino-acid permeases. Mutants lacking one of the functional subunits of the amine dehydrogenase (*ΔqhpA)* or *Δfae3* showed no growth defect on C_1_-compounds. *M. universalis* FAM5 strains with a lesion in the H_4_-folate pathway were not able to use any C_1_-compound, methanol or methylamine. Genes essential for C_1_-assimilation (the serine cycle and glyoxylate shunt) and H_4_MTP-pathway for formaldehyde oxidation showed similar levels of expression on both C_1_-carbon sources. *M. universalis* FAM5 possesses three homologs of the formaldehyde activating enzyme, a key enzyme of the H_4_MTP-pathway. Strains lacking the canonical Fae (*fae1*) lost the ability to grow on both C_1_-compounds. However, upon incubation on methylamine the *fae1*-mutant produced revertants (*Δfae1^R^*), which regained the ability to grow on methylamine. Double and triple mutants (*Δfae1^R^Δfae3*, or *Δfae1^R^Δfae2* or *Δfae1^R^Δfae2Δfae3)* constructed in the revertant strain background showed growth similar to the *Δfae1^R^* phenotype. The metabolic pathways for utilization of methanol and methylamine in *Methyloversatilis universalis* FAM5 are reconstructed based on these gene expression and phenotypic data.

## 1. Introduction

Facultative betaproteobacterial methylotrophs, belonging to *Burkholderiales* and *Rhodocyclales*, occupy a variety of ecological niches, including soils, sediments, plant biomass, wastewater sludge, hot springs, and oil sands [1,2,3,4,5,6,7]. These bacterial lineages show exceptionally versatile metabolic capabilities, including the ability to utilize single carbon compounds, such as methanol and methylated amines [8,9,10]. Despite the relatively high abundance of these facultative methylotrophic betaproteobacteria in nature, only a few of them have been isolated in pure culture and characterized [4,5,6,9,10]. C_1_-utilization pathways have been investigated in *Methyloversatilis universalis* and *Methyloversatilis thermotolerans* (both are members of the family *Rhodocyclaceae*) and *Methylibium petroleiphilum* PM1 (a representative of unclassified *Burkholderiales*) [11,12,13]. It has been shown that a homolog of the pyrroloquinoline quinone (PQQ)-dependent ethanol dehydrogenase is essential for oxidation of C_1_ and C_2_ alcohols during aerobic or anaerobic growth in *M. universalis* [8,13]. The same enzyme is crucial for methanol utilization even in strains possessing the canonical methanol dehydrogenase, such as *Methyloversatilis* sp. 18–153 or RZ94 [8,10].

In addition to methanol, all tested members of the genera *Methyloversatilis* are capable of utilizing methylated amines [6,10]. *M. universalis* FAM5 oxidizes methylamine via the *N*-methylglutamate pathway [12]. The pathway includes three enzymes: the *N*-methylglutamate synthase (NMGS, encoded by *mgsABC* gene cluster), which can produce *N*-methylglutamate from glutamate and methylamine; the *N*-methylglutamate dehydrogenase (NMGDH, encoded by *mgdABCD* gene cluster), which is predicted to produce methylene-tetrahydrofolate (H_4_F) and regenerated glutamate from *N*-methylglutamate; and the gamma-glutamylmethylamine synthetase (GMAS, *gmas*), a reversible enzyme which produces gamma-glutamylmethylamine (GMA) from glutamate and methylamine using ATP. The gamma-glutamylmethylamine synthetase is predicted to serve as the first enzyme of the pathway, however the exact functional role of the enzyme and its product, gamma-glutamylmethylamine, are not well defined. It has been predicted that the enzyme contributes to methylamine detoxification, by converting the potentially toxic amine into a metabolically neutral form [14]. This vision of the pathway has been supported by a number of studies, including mutagenesis, ^13^C-labeling and *in vivo* NMR investigation [12,14,15,16]. The GMA has also been proposed to be a substrate for NMGS instead of methylamine [17,18,19]. Overall, the exact topology of the pathway is still awaiting a careful enzymatic investigation. Nonetheless, several genomic studies suggest that the *N*-methylglutamate pathway is recruited for methylamine utilization by a number of methylotrophic and non-methylotrophic bacteria [16,17,18,19,20,21,22].

Recently the genomes of seven strains representing three *Methyloversatilis* species have been sequenced at the University of Washington [23] and in collaboration with the Joint Genomic Institute (JGI) [10]. The C_1_-pathways were found to be highly conserved in all strains sequenced. It was predicted that formaldehyde, a product of methanol or methylamine oxidation could be converted to formate by a tungsten-dependent aldehyde dehydrogenase in addition to the tetrahydrofolate (H_4_F) or tetrahydromethanopterin (H_4_MPT)-pathways [5,13,23]. Formate is further oxidized to CO_2_ by two NAD-dependent formate dehydrogenases [13]. Two C_1_-assimilation pathways, the serine cycle and Calvin-Benson-Bassham (CBB) cycle, could be predicted for sequenced *Methyloversatilis spp*, with the exception of *M. thermotolerans* 3t^T^, which possesses only the serine cycle [10,11,23]. Only serine cycle enzymes were induced in *M. universalis* cells during anaerobic growth on methanol [13]. Genome analyses also indicate that *M. universalis* FAM5 also possesses a heme-containing amine dehydrogenases (Qhp). It has been shown that the Qhp oxidizes a number of aliphatic and aromatic amines [24,25,26]. Qhp was highly expressed during growth of the *Paracoccus denitrifican*s IFO 12442 on methylamine. However, the enzyme displays relatively low affinity for methylamine and is specific to *n*-butylamine [25]. It has been speculated that during growth of *P. denitrifican*s on methylamine the enzyme plays a secondary role and might contribute to oxidation/detoxification of the amine [25,26]. The contribution of the enzyme to metabolism of methylamine has not been tested by mutagenesis.

Here we investigate pathways for aerobic utilization of methanol and methylamine in *M. universalis* FAM5. The predicted end-product of methylamine oxidation via the *N*-methylglutamate pathways is methylene-H_4_F [12]. Thus the metabolic arrangement of methylamine utilization was expected to differ from the methanol utilization network, where formaldehyde is the key end product of the methanol oxidation [8].

## 2. Materials and Methods

### 2.1. Bacterial Strains, Plasmids and Culture Conditions

The *M. universalis* sp FAM5 strains used in this study are listed in Table 1. Since the wild type aggregates in liquid culture we selected a strain of the *M. universalis* (named here FAM5^E^) that does not clump. No differences in growth rate between strain FAM5 and FAM5^E^ on plates or in liquid culture were observed (data not shown). The main advantage of the strain FAM5^E^ is that it produces reliable reading of optical density and does not require homogenization. The strain FAM5^E^ was used to generate and test the majority of mutants described in this study (Table 1).

**Table 1:** Growth phenotypes of the *Methyloversatilis universalis* wild type and mutant strains.

Strain	Description	Succinate	Methanol	Methylamine	Succinate + C_1_ *
FAM5	Wild type, forms aggregates in liquid culture, Kan^S^Tet^S^Cm^R^	++++	+++	++++	++++
FAM5^E^	Trait of FAM5 which does not form aggregates in liquid culture, Kan^S^Tet^S^Cm^R^	++++	+++	++++	++++
*ΔmgdD*	FAM5-*ΔmgdD:kan*, Kan^R^Tet^S^Cm^R^	++++	+++	-	NT
*ΔmgsC*	FAM5-*ΔmgsC:kan*, Kan^R^Tet^S^Cm^R^	++++	+++	-	NT
*Δfae1*	FAM5^E^-*Δfae1:kan*, Kan^R^Tet^S^Cm^R^	++++	-	-	NT
*Δfae2*	FAM5^E^-*Δfae2:kan*, Kan^R^Tet^S^Cm^R^	++++	+++	++++	NT
*Δfae3*	FAM5^E^-*Δfae3:kan*, Kan^R^Tet^S^Cm^R^	++++	+++	++++	NT
*ΔmtdA*	FAM5^E^-*ΔmtdA:kan*, Kan^R^Tet^S^Cm^R^	++++	-	-	+++ ^(1)^
*ΔqhpA*	FAM5-*ΔqhpA*, Kan^S^Tet^S^Cm^R^	++++	+++	++++	NT
*Δfae1ΔqhpA*	FAM5-*ΔqhpAΔfae1:kan*, Kan^R^Tet^S^Cm^R^	++++	-	++	NT
*Δfae2ΔqhpA*	FAM5-*ΔqhpAΔfae2:kan*, Kan^R^Tet^S^Cm^R^	++++	+++	++++	NT
*Δfae3ΔqhpA*	FAM5-*ΔqhpAΔfae3:kan*, Kan^R^Tet^S^Cm^R^	++++	+++	++++	NT
*Δfae1^R^ΔqhpAΔfae3*	FAM5^E^-*ΔqhpA Δfae1Δfae3:kan*, Kan^R^Tet^S^Cm^R^	++++	-	-	NT
*Δfae1^R^*	Revertant of FAM5^E^-*Δfae1*, Kan^S^Tet^S^Cm^R^	++++	-	++	++++ ^(2)^
*Δfae1Δfae3*	FAM5-*Δfae1Δfae3:kan*, Kan^R^Tet^S^Cm^R^	++++	-	-	NT
*Δfae1^R^Δ fae3*	FAM5-*Δfae1Δfae3*, Kan^S^Tet^S^Cm^R^	++++	-	++	++++ ^(2)^
*Δfae1Δ fae2*	FAM5-*Δfae1Δfae2:kan*, Kan^R^Tet^S^Cm^R^	++++	+++	++++	NT
*Δfae3*	FAM5^E^-*Δfae3*, Kan^S^Tet^S^Cm^R^	++++	+++	++++	NT
*Δfae3Δfae2*	FAM5^E^-*Δfae3 Δfae2:kan*, Kan^R^Tet^S^Cm^R^	++++	+++	++++	NT
*Δfae1Δfae2Δfae3*	FAM5^E^-*Δfae1Δfae3Δfae2:kan*, Kan^R^Tet^S^Cm^R^	++++	-	-	NT

NT, not tested; “++++” correspond to doubling time of 6 h; “+++” correspond to doubling time of 9 h; “++” correspond to 12 h doubling time; and “-” indicates no growth. Methanol (5 mM) was added to test if a mutant strain is sensitive to formaldehyde ^(2)^ or unable to produce methyl-H_4_folate for purine synthesis ^(1)^.

Cells were grown in previously described minimal medium [6] with succinate (20 mM), methylamine (30 mM or 5 mM), and methanol (25 mM) as growth substrates. The following antibiotic concentrations were used for *M. universalis*: tetracycline (Tet), 1.0 mg L^−1^; kanamycin (Kan), 100 mg L^−1^; and chloramphenicol (Cmp), 10–15 mg L^−1^.

The following cloning vectors were used: pCR2.1 (Invitrogen, Carlsbad, CA, USA) for cloning of PCR products, pCM184 for generation of single, double and triple mutant strains, and the pCM157 system for construction of unmarked mutations (Table 2) [27]. *Escherichia coli* strains were routinely cultivated at 37 °C in Luria-Bertani medium (BD Difco, Franklin Lakes, NJ, USA). When indicated, antibiotic concentrations were used: Tet, 12.5 mg L^−1^; Kan, 100 mg L^1^; and ampicillin (Amp), 100 mg L^−1^.

**Table 2:** Bacterial strains and plasmids used in this study.

Strain/Plasmid	Markers/Description	Reference/Source
*Escherichia coli* One Shot TOP 10	F-*mcr*A* Δ* (*mrr-hsd*RMS*-mcr*BC) Φ80 *lac*Z*Δ*M15 *Δlac*X74 *rec*A1 *ara*D139 *Δ (ara-leu)*7697 *gal*U *gal*K *rps*L Str^R^ *end*A1 *nup*G	Invitrogen
*Escherichia coli* S17-1	*rec*A *pro hsd*R RP4-2-Tc:Mu-Km:Tn7 Tmp^R^ Spc^R^Str^R^	[28]
pCR2.1	Kan^R^, Amp^R^	Invitrogen
pDrive	Kan^R^, Amp^R^	Qiagen
pCM184	Broad-host-range allelic exchange vector, Kan^R^, Tet^R^	[27]
pCM157	Cre/LoxP, Tet^R^	[27]
pMgdD	pCM184 with *mgdD* upstream and downstream flanks	This study
pMgsC	pCM184 with *mgsC* upstream and downstream flanks	This study
pFAE1	pCM184 with *fae1* upstream and downstream flanks	This study
pFAE2	pCM184 with *fae2* upstream and downstream flanks	This study
pFAE3	pCM184 with *fae3* upstream and downstream flanks	This study
pQHNDH1	pCM184 with *qhpA* upstream and downstream flanks	This study
pQHNDH2	pCM184 with *qhpX* upstream and downstream flanks	This study
pMtdA	pCM184 with *mtdA* upstream and downstream flanks	This study

### 2.2. DNA Manipulations

DNA was isolated using the QIAamp DNA Mini kit (Qiagen, Venlo, Netherlands). Plasmid DNA was purified using the Qiagen Miniprep Kit (Qiagen). *E. coli* transformation, restriction enzyme digestion and ligation reactions were carried out as described by Sambrook *et al.* [29]. Polymerase chain reaction (PCR) amplifications were performed using hot-start *Taq* polymerase (Qiagen) in accordance with the manufacturer’s instructions. Primers used for PCR amplification of upstream or downstream regions (approximately 450–600 bp) of each targeted gene are listed in Table S1. Amplified fragments were cloned into pCR2.1, sequenced and then sub-cloned into pCM184 using appropriate restriction sites. After verification of the nucleotide sequence, the plasmids were transformed into *E. coli* S17-1, and the resulting donor strains were mated with wild-type *M. universalis* FAM5 via biparental mating as previously described [8]. The identity of the double-crossover mutants was verified by diagnostic PCR with primers specific to the insertion sites. Mutant phenotypes were assessed on solid media and in liquid culture with succinate, pyruvate, methanol or methylamine as carbon sources.

### 2.3. RNA-seq Experiments

Trascriptomic experiments were carried out with *M. universalis* strain FAM5. Cells pre-grown on methanol (10 mM) were used for inoculation of methanol (25 mM) or methylamine (35 mM) cultures at 5:100 ratios (inoculum:fresh media). Cultures were grown to mid-exponential phase (OD_600_ 0.4 ± 0.05). The cellular activities were terminated by addition of “stop solution” as described [30]. The cells were collected by centrifugation at 4300× *g* at 4 °C for 10 min. mRNA samples were isolated and enriched as previously described [30] and submitted to the University of Washington’s High-Throughput Sequencing Solutions Center on dry ice for single-read Illumina^®^ sequencing (Department of Genome Sciences, University of Washington; http://www.htseq.org/). The RNA-Seq sequence data sets ranged from 12.19 to 25.46 million reads (36 bp) per sample. The *M. universalis* FAM5 genome to be used as the alignment scaffold was downloaded from MaGE (https://www.genoscope.cns.fr/agc/microscope/mage/) on 31 March 2014. The raw reads were aligned to the scaffold using BWA version 0.7.4-r385 with default parameters [31]. The alignments were post-processed and sorted into BAM files with SAMTools version 0.1.19-44428cd [32]. Reads were attributed to open reading frames (ORFs) using the htseq-count tool from the “HTSeq” framework version 0.5.4p5 in the “intersection-nonempty” mode [33]. The ratios of reads mapped per ORF in the two conditions were calculated along with *p*-values using DESeq2 [33]. The *p*-values in Table 2 were corrected for multiple testing using the *q*-value method of Storey [34]. Proportions of reads mapped to rRNA were 78%–90% for RNA-libraries prepared from cells grown on methanol, and 91%–92% for RNA-libraries prepared from cells grown on methylamine. For display and presentation purposes, the Reads per Kilobase per Million reads sequenced (RPKM) were calculated [35].

### 2.4. Enzyme Assays

All cell extracts (50 mL) for activity were prepared from methylamine-grown cultures. Cells were harvested by centrifugation at 4500× *g* using a Sorvall RC-5B centrifuge at 4 °C for 10 min. The supernatant was removed and the cell pellet was stored at −80 C. Cell pellets were thawed and resuspended in 1 mL of 100 mM potassium phosphate buffer, pH 7.6 or 7.2 and broken using a French press (three times, 1000 psi). The extracts were centrifuged at 28,000× *g* for 5 min to remove cell debris. Qhp activity was detected by measuring amine dehydrogenase activity using a spectrophotometric assay measuring the reduction rate of potassium ferricyanide (500 μM, at 420 nm) in the presence of cell free extract (100 μg), and methylamine (25 mM) as substrate at room temperature. The extinction coefficient of potassium ferricyanide is 1.02 mM^−1^ cm^−1^ at pH = 7.6. The total volume of the reaction was 200 μL. Activity was measured using a Spectramax 190 plate reader (Molecular Devices, Sunnyvale, CA, USA); a minimum of two biological replicates was assayed.

### 2.5. Accession Numbers

The RNA-seq data were uploaded into NCBI/GEO under accession number GSE63822.

## 3. Results

### 3.1. Gene Expression Profiles: Methanol *vs.* Methylamine

Whole transcriptome shotgun sequencing data were generated for *M. universalis* cells grown on methanol or methylamine using a high-throughput sequencing (Illumina) platform. Based on relative expression, genes (omitting rRNAs*)* could be grouped into six major categories: genes with very high (Reads per Kilobase Million (RPKM) ≥ 10000), high (RPKM ≥ 5000), moderate (5000 > RPKM ≥ 1000), modest (1000 > RPKM ≥ 250), and low (250 > RPKM ≥ 50) expression, and not expressed (RPKM < 50). Annotation of the *M. universalis FAM5* genome predicts 4027 coding sequences [23]. Based on the expression data, 950 (24%) genes were not expressed under any condition tested*.* Among the silent functional modules were genes encoding denitrification reactions (nitrate and nitrite reductases, nitrate transporters), phototrophy (light-harvesting complexes) and pathways for utilization of urea, acetoin, methanesulfonate, and phenolic compounds. The majority of genes fell into low expression categories (45%). About 25% of genes displayed modest expression, 5% of genes showed moderate expression and only a small fraction (1%) of the genome showed very high/high expression.

Most of the previously recognized metabolic modules essential for oxidation of C_1_-compounds fell into very high to moderate categories. Expression of central metabolic pathways, such as the citric acid cycle, amino acid biosynthesis, gluconeogenesis and energy generating pathway (respiratory chain and ATP-synthase) genes remained mostly unchanged (Table 3). Genes encoding the H_4_MPT synthesis and the H_4_MPT-linked C_1_-transfer enzymes and the serine cycle enzymes also remained unchanged, suggesting that these pathways contribute in a similar fashion to methanol and methylamine utilization (Table 3).

**Figure 1:**
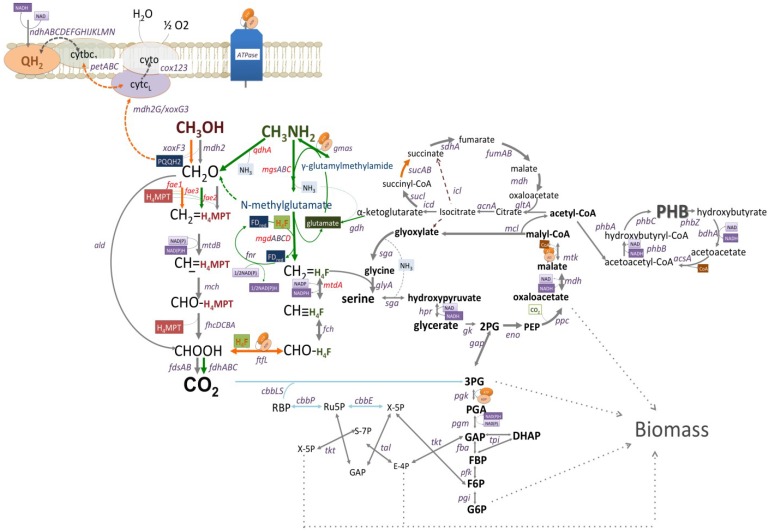
The reconstructed pathway of methanol and methylamine utilization in *Methyloversaltilis universalis* FAM5 based on genomic, transcriptomic and mutagenesis data. Genes mutated in this study are shown in red. Pathways upregulated (6–11 fold) by growth on methylamine are shown in green; Pathways slightly downregulated (1.5–3 fold) by growth on methylamine are shown in orange; Grey lines show pathways whose expression do not change; and Blue lines indicate steps that are not expressed on tested C1-compounds, methanol or methylamine.

Cells growth on methanol showed a slightly higher (about 1.4 fold) abundance of transcripts for methanol dehydrogenase (*mdh2*) and the associated cytochrome gene (*cyt*) (Table 3; Figure 1). The relative abundance of formate-tetrahydrofolate ligase/formyltetrahydrofolate synthetase (*ftfL*) transcripts, a tungsten-containing aldehyde ferredoxin oxidoreductase, a distant homolog of the PQQ-dependent methanol dehydrogenase (*xoxF3G3J3*), cytochrome bc_1_ reductase complex, a putative NADP(H)-dependent aldo/keto reductase and a number of hypothetical proteins were higher (1.6–3 folds) in methanol grown cells (Table 3; Figure 1). Genes encoding the *N*-methylglutamate pathway enzymes, co-clustered conserved proteins and a putative transcriptional regulator, were up-regulated (6−10-fold) during growth on methylamine (Table 3, Figure 1). Among other genes altered by the shift to methylamine were: a seven gene cluster (*qhpRADCBFE*) encoding a heme-containing amine dehydrogenase (Qhp, *qhpADC*), and associated proteins involved in the posttranslational modification and regulation; a ferredoxin; and a distant homolog of the formaldehyde activating enzyme (*fae3*). The fae3 gene was almost 10 fold higher in cells shifted to methylamine.

**Table 3:** Gene expression profile in methane or methylamine grown cells of *M. universalis* FAM5.

Gene ID (Old)	Gene ID (New)	Gene Product	Methanol (RPKM) *	Methylamine (RPKM) *	Fold Change	*q*-Value
*Methylamine oxidation*						
METUNv1_760110	METUNv2_580110	Gmas, Gamma glutamylmethylamide synthetase	2961.80	19,440.25	6.56	0.65
METUNv1_760111	METUNv2_580111	MgsA, *N*-methyl glutamate synthase subunit A	2727.43	22,221.30	8.15	0.73
METUNv1_760112	METUNv2_580112	MgsB, *N*-methyl glutamate synthase subunit B	2680.76	19,852.55	7.41	0.69
METUNv1_760113	METUNv2_580113	MgsC, *N*-methyl glutamate synthase subunit C	4221.56	53,956.55	12.78	0.67
METUNv1_760114	METUNv2_580114	MgdA, *N*-methyl glutamate dehydrogenase/oxidoreductase subunit A	1414.90	13,014.45	9.20	0.74
METUNv1_760115	METUNv2_580115	MgdB, *N*-methyl glutamate dehydrogenase/oxidoreductase subunit B	388.00	3906.31	10.07	0.74
METUNv1_760116	METUNv2_580116	MgdC, *N*-methyl glutamate dehydrogenase/oxidoreductase subunit C	3389.10	26,611.05	7.85	0.75
METUNv1_760117	METUNv2_580117	MgdD, *N*-methyl glutamate dehydrogenase/oxidoreductase subunit D	701.94	7962.74	11.34	0.71
METUNv1_760127	METUNv2_580127	QhpB, Quinohemoprotein amine dehydrogenase, beta subunit	420.06	4164.18	9.91	0.68
METUNv1_760128	METUNv2_580128	QhpA, Quinohemoprotein amine dehydrogenase, alpha subunit	733.76	7390.84	10.07	0.65
METUNv1_760130	METUNv2_580130	QhpC, Quinohemoprotein amine dehydrogenase, SAM-radical dependent activating subunit	380.86	4283.06	11.25	0.00
*Methanol oxidation*						
METUNv1_770214	METUNv2_590217	XoxF1, PQQ-linked dehydrogenase	2023.28	3380.16	1.67	0.50
METUNv1_770216	METUNv2_590218	XoxF2, PQQ-linked dehydrogenase	10,962.17	16,741.70	1.53	0.05
METUNv1_590046	METUNv2_420045	XoxF3, PQQ-dependent dehydrogenase	1213.63	659.01	−1.84	0.62
METUNv1_590042	METUNv2_420041	XoxJ3, Extracellular solute-binding protein family 3	4399.57	1403.51	−3.13	0.05
METUNv1_590043	METUNv2_420042	XoxG3, Cytochrome c class I	3156.11	1089.89	−2.90	0.30
METUNv1_590049	METUNv2_420048	Mdh2, PQQ-dependent methanol/ethanol dehydrogenase	48,100.35	34,793.20	−1.38	0.77
METUNv1_590050	METUNv2_420049	Mdh2J, Extracellular solute-binding protein family	2486.88	903.00	−2.75	0.69
METUNv1_590051	METUNv2_420050	Mdh2G, cytochrome c-type protein	1732.91	595.39	−2.91	0.58
*Formaldehyde oxidation*						
METUNv1_580096	METUNv2_410093	Fae 1, Formaldehyde-activating enzyme	18,235.00	11,635.85	−1.57	0.82
METUNv1_660037	METUNv2_480039	Fae 2, Formaldehyde-activating enzyme	561.52	642.50	1.14	0.89
METUNv1_700516	METUNv2_520523	Fae 3, Formaldehyde activating enzyme	584.51	6861.86	11.74	0.00
METUNv1_590006	METUNv2_420006	Orf 9, Involved in biosynthesis of tetrahydromethanopterin. Essential for formaldehyde oxidation.	430.06	187.51	−2.29	0.21
METUNv1_580095	METUNv2_410092	Orf 7, Involved in tetrahydromethanopterin-linked formaldehyde oxidation.	371.25	549.26	1.48	0.75
METUNv1_580094	METUNv2_410091	Orf 5, Involved in biosynthesis of tetrahydromethanopterin	489.69	493.89	1.01	0.93
METUNv1_580093	METUNv2_410090	Mch, Methenyltetrahydromethanopterin cyclohydrolase	620.26	736.89	1.19	0.87
METUNv1_580092	METUNv2_410089	OrfY, Involved in tetrahydromethanopterin C1 transfer.	896.40	819.12	−1.09	0.94
METUNv1_580091	METUNv2_410088	MtdB, NAD-dependent methylenetetrahydromethanopterin dehydrogenase	1295.29	1379.77	1.07	0.88
METUNv1_580088	METUNv2_410086	FhcB, Formyltransferase/hydrolase complex subunit B	587.63	515.00	−1.14	0.92
METUNv1_580087	METUNv2_410085	FhcA, Formyltransferase/hydrolase complex subunit A	766.77	744.38	−1.03	0.93
METUNv1_580086	METUNv2_410084	FhcD, Formyltransferase/hydrolase complex subunit D	598.76	547.64	−1.09	0.94
METUNv1_580085	METUNv2_410083	FhcC, Formyltransferase/hydrolase complex subunit C	634.50	467.32	−1.36	0.88
METUNv1_590040	METUNv2_420039	Ald, Tungsten-containing aldehyde ferredoxin oxidoreductase	408.13	155.57	−2.62	0.17
METUNv1_490013	METUNv2_320014	AldB, Aldehyde dehydrogenase B	2762.93	2224.63	−1.24	0.94
*Formate oxidation*						
METUNv1_770385	METUNv2_590384	Fdh, Putative formate dehydrogenase subunit A	634.02	668.94	1.06	0.92
METUNv1_700257	METUNv2_520260	FdhD, NAD-linked formate dehydrogenase delta subunit	38.00	76.63	2.02	0.55
METUNv1_700258	METUNv2_520261	FdhC, Formate dehydrogenase, accessory protein	751.50	1024.07	1.36	0.74
METUNv1_700259	METUNv2_520262	FdhA, NAD-dependent formate dehydrogenase alpha subunit	3720.44	5479.72	1.47	0.39
METUNv1_700260	METUNv2_520263	FdhB, NAD-dependent formate dehydrogenase beta subunit	2758.00	4434.84	1.61	0.46
METUNv1_700261	METUNv2_520264	FdhG, NAD-dependent formate dehydrogenase gamma subunit	1127.75	1882.41	1.67	0.67
METUNv1_700262	METUNv2_520265	FdhR, Formate dehydrogenase regulator	210.25	231.00	1.10	0.90
METUNv1_570005	METUNv2_400021	FdsA, NAD-dependent, tungsten-containing formate dehydrogenase alpha subunit	1438.46	1527.51	1.06	0.91
METUNv1_570006	METUNv2_400022	FdsB, NAD-dependent, tungsten-containing formate dehydrogenase beta subunit	951.26	1018.18	1.07	0.90
*H_4_F-pathway/Serine cycle/Glyoxylate shunt*						
METUNv1_460318	METUNv2_290319	FtfL, formate-tetrahydrofolate ligase/synthetase	4729.66	1674.32	−2.82	0.54
METUNv1_460309	METUNv2_290310	Fch, Methenyltetrahydrofolate cyclohydrolase	2091.39	1541.64	−1.36	0.90
METUNv1_460310	METUNv2_290311	MdtA, NADP-dependent methylenetetrahydrofolate dehydrogenase	4363.44	2973.78	−1.47	0.93
METUNv1_460311	METUNv2_290312	Hpr, Hydroxypyruvate reductase, NAD(P)H-dependent.	3620.17	3220.89	−1.12	0.89
METUNv1_460312	METUNv2_290313	Sga, Serine-glyoxylate aminotransferase	10,590.93	8570.41	−1.24	0.79
METUNv1_460313	METUNv2_290314	GlyA, Serine hydroxymethyltransferase	6313.10	5292.54	−1.19	0.87
METUNv1_460314	METUNv2_290315	MtkA, Malate thiokinase large subunit	4955.01	6292.07	1.27	0.73
METUNv1_460315	METUNv2_290316	MtkB, Malate thiokinase small subunit	5081.45	5363.84	1.06	0.73
METUNv1_460316	METUNv2_290317	Ppc1, phosphoenolpyruvate carboxylase	2661.63	2768.19	1.04	0.86
METUNv1_460317	METUNv2_290318	Mcl, malyl-CoA lyase	3260.85	4275.94	1.31	0.58
METUNv1_770329	METUNv2_590331	Gk, Glycerate kinase	1197.01	1116.38	−1.07	0.93
METUNv1_770169	METUNv2_590170	Ppc2, Phosphoenolpyruvate carboxylase	1034.15	1074.00	1.04	0.91
METUNv1_460302	METUNv2_290303	Eno, Enolase	2095.13	1936.51	−1.08	0.92
METUNv1_710053	METUNv2_530053	Pgm, Phosphoglyceromutase	470.57	517.14	1.10	0.90
METUNv1_620020	METUNv2_450021	Ms, Malate synthase A	353.94	269.76	−1.31	0.89
METUNv1_620018	METUNv2_450018	Icl, Isocitrate lyase	12,174.92	8389.98	−1.45	0.85
*CO_2_ Fixation (CBB cycle)*						
METUNv1_750044	METUNv2_570044	CbbR, RuBisCO operon transcriptional regulator	195.62	189.12	−1.03	0.93
METUNv1_750045	METUNv2_570045	CbbL, Ribulose-1,5-bisphosphate carboxylase large subunit	127.63	130.88	1.03	0.94
METUNv1_750046	METUNv2_570046	CbbS, Ribulose bisphosphate carboxylase small subunit	41.62	47.50	1.14	0.92
METUNv1_750047	METUNv2_570047	CbxX, chromosomal AAA type ATPase	8.75	11.94	1.36	0.89
METUNv1_750048	METUNv2_570048	CbbY, haloacid dehalogenase	7.25	14.56	2.01	0.73
METUNv1_750049	METUNv2_570049	CbbE, Ribulose-phosphate 3-epimerase	13.88	16.13	1.16	0.91
METUNv1_750050	METUNv2_570050	Pgp, phosphoglycolate phosphatase	12.50	13.00	1.04	0.94
METUNv1_750051	METUNv2_570051	CbbF, Fructose-1,6-bisphosphatase	26.75	30.51	1.14	0.92
METUNv1_750052	METUNv2_570052	CbbP, Phosphoribulokinase	26.69	32.00	1.20	0.92
METUNv1_750053	METUNv2_570053	CbbT, Transketolase	24.88	42.56	1.71	0.73
METUNv1_750054	METUNv2_570054	CbbG, Glyceraldehyde-3-phosphate dehydrogenase	17.50	19.56	1.12	0.93
METUNv1_750055	METUNv2_570055	CbbA, Fructose-bisphosphate aldolase	72.75	65.50	−1.11	0.93
METUNv1_700111	METUNv2_520114	CbbR, RuBisCO operon transcriptional regulator	360.50	255.84	−1.41	0.90
METUNv1_760018	METUNv2_580018	CbbQ, Post-translational RubisCO activator	16.25	19.38	1.19	0.91
Sugar Phosphate Interconversions						
METUNv1_470279	METUNv2_300282	Fbp, Fructose-1,6-bisphosphatase	403.90	440.81	1.09	0.91
METUNv1_700104	METUNv2_520106	Fba, Fructose-bisphosphate aldolase	920.68	1094.83	1.19	0.85
METUNv1_700105	METUNv2_520107	Pyk, Pyruvate kinase II	807.00	786.28	−1.03	0.92
METUNv1_700106	METUNv2_520108	Pgk, Phosphoglycerate kinase	693.63	684.62	−1.01	0.92
METUNv1_700107	METUNv2_520109	Gapdh, Glyceraldehyde 3-phosphate dehydrogenase	1478.94	1536.51	1.04	0.90
METUNv1_700108	METUNv2_520110	Tk, Transketolase	982.15	730.45	−1.34	0.89
METUNv1_700109	METUNv2_520111	Prk, Phosphoribulokinase	380.94	483.87	1.27	0.82
METUNv1_700110	METUNv2_520112	Fbp3, Fructose-1,6-bisphosphatase	902.64	846.62	−1.07	0.94
METUNv1_470090	METUNv2_300091	Pps, Phosphoenolpyruvate synthase	1610.66	1360.78	−1.18	0.94
METUNv1_460093	METUNv2_290089	Pck, Phosphoenolpyruvate carboxykinase (GTP)	242.99	191.39	−1.27	0.89
METUNv1_580038	METUNv2_410037	Pfk, Pyrophosphate-dependent phosphofructokinase	866.70	1075.27	1.24	0.85
METUNv1_580036	METUNv2_410036	Pyrophosphate-energized inorganic pyrophosphatase	747.52	887.26	1.19	0.88
METUNv1_580049	METUNv2_410048	Pyrophosphate phosphohydrolase	555.12	602.37	1.09	0.89
*TCA cycle*						
METUNv1_700127	METUNv2_520130	E1 component of pyruvate dehydrogenase	959.88	1210.51	1.26	0.80
METUNv1_700126	METUNv2_520129	E2 component of pyruvate dehydrogenase	217.00	302.25	1.39	0.77
METUNv1_700125	METUNv2_520128	E3 component of pyruvate dehydrogenase	482.26	555.08	1.15	0.88
METUNv1_700186	METUNv2_520190	Succinyl-CoA synthetase beta subunit	705.14	556.31	−1.27	0.91
METUNv1_700187	METUNv2_520191	Succinyl-CoA synthetase alpha subunit	622.44	414.43	−1.50	0.86
METUNv1_470127	METUNv2_300128	Fumarate hydratase class I	659.82	590.00	−1.12	0.93
METUNv1_460167	METUNv2_290164	Fumarate hydratase class II (fumarase C)	185.50	141.26	−1.31	0.91
METUNv1_460003	METUNv2_290002	ME1, Malic Enzyme	952.12	789.15	−1.21	0.93
METUNv1_520009	METUNv2_350011	Mdh, Malate dehydrogenase	2673.56	1934.79	−1.38	0.91
METUNv1_520011	METUNv2_350013	Succinate dehydrogenase cytochrome b556 subunit	324.52	191.00	−1.70	0.70
METUNv1_520012	METUNv2_350014	Succinate dehydrogenase anchor subunit	572.13	412.89	−1.39	0.88
METUNv1_520013	METUNv2_350015	Succinate dehydrogenase flavoprotein subunit	4086.04	2492.77	−1.64	0.84
METUNv1_520014	METUNv2_350016	Succinate dehydrogenase Fe–S protein	843.00	709.16	−1.19	0.93
METUNv1_520016	METUNv2_350018	Citrate synthase	2249.29	2218.66	−1.01	0.91
METUNv1_520017	METUNv2_350019	E1 component of alpha-ketoglutarate dehydrogenase	1429.24	1533.03	1.07	0.89
METUNv1_520018	METUNv2_350020	E2 component of alpha-ketoglutarate dehydrogenase	697.75	762.99	1.09	0.90
METUNv1_520019	METUNv2_350021	E3 component of alpha-ketoglutarate dehydrogenase	1364.02	1401.04	1.03	0.89
METUNv1_620012	METUNv2_450011	Isocitrate dehydrogenase kinase/phosphatase	241.63	308.13	1.28	0.84
METUNv1_620013	METUNv2_450012	Isocitrate dehydrogenase (NADP+)	2578.02	2899.29	1.12	0.80
*Amino Acid Synthesis*						
METUNv1_660052	METUNv2_480054	Glutamate synthase (NADPH) large chain (NADPH-GOGAT)	3985.94	2866.80	−1.39	0.87
METUNv1_660053	METUNv2_480055	Glutamate synthase (NADPH) small chain (NADPH-GOGAT)	1357.89	903.01	−1.50	0.91
METUNv1_450044	METUNv2_280044	Glutamine synthetase (Glutamate-ammonia ligase)	2361.78	2288.13	−1.03	0.56
METUNv1_470103	METUNv2_300104	Glutamate dehydrogenase, NADP-specific (NADP-GDH)	1040.15	1519.76	1.46	0.93
METUNv1_470104	METUNv2_300105	Aspartate aminotransferase (Transaminase A) (AspAT)	1020.04	941.84	−1.08	0.92
METUNv1_470184	METUNv2_300183	Putative aspartate transaminase	617.88	620.11	1.00	0.93
METUNv1_750043	METUNv2_570043	Serine-pyruvate aminotransferase	679.01	606.13	−1.12	0.91
*Oxidative Phosphorylation*						
METUNv1_750182	METUNv2_570182	ATP synthase F0, A chain	886.37	965.57	1.09	0.83
METUNv1_750183	METUNv2_570183	ATP synthase F0, C chain	1218.52	1365.89	1.12	0.93
METUNv1_750184	METUNv2_570184	ATP synthase F0, B chain	2528.92	2775.88	1.10	0.89
METUNv1_750185	METUNv2_570185	ATP synthase delta chain	2885.17	2564.77	−1.12	0.86
METUNv1_750186	METUNv2_570186	ATP synthase subunit alpha subunit	8915.44	6780.46	−1.31	0.89
METUNv1_750187	METUNv2_570187	ATP synthase gamma subunit	5294.58	4705.58	−1.13	0.93
METUNv1_750188	METUNv2_570188	ATP synthase beta subunit	9593.01	6768.24	−1.42	0.92
METUNv1_750189	METUNv2_570189	ATP synthase epsilon subunit	1784.40	1464.79	−1.22	0.90
METUNv1_770336	METUNv2_590337	NAD(P) transhydrogenase subunit alpha	858.91	858.01	−1.00	0.93
METUNv1_770337	METUNv2_590338	NAD(P) transhydrogenase subunit beta	399.38	455.25	1.14	0.92
METUNv1_460126	METUNv2_290122	NADH-quinone oxidoreductase chain A	156.74	148.38	−1.06	0.93
METUNv1_460127	METUNv2_290123	NADH-quinone oxidoreductase subunit B	451.50	376.26	−1.20	0.94
METUNv1_460128	METUNv2_290124	NADH (or F420H2) dehydrogenase subunit C	388.51	353.76	−1.10	0.94
METUNv1_460129	METUNv2_290125	NADH-ubiquinone oxidoreductase D subunit	754.27	681.62	−1.11	0.92
METUNv1_460130	METUNv2_290126	NADH-quinone oxidoreductase subunit E	207.95	211.51	1.02	0.92
METUNv1_460131	METUNv2_290127	NADH-quinone oxidoreductase subunit F	628.76	528.76	−1.19	0.87
METUNv1_460132	METUNv2_290128	NADH-quinone oxidoreductase subunit G	1248.13	938.27	−1.33	0.93
METUNv1_460133	METUNv2_290129	NADH-quinone oxidoreductase subunit H	520.13	381.75	−1.36	0.93
METUNv1_460134	METUNv2_290130	NADH-quinone oxidoreductase subunit I	327.57	337.82	1.03	0.94
METUNv1_460135	METUNv2_290131	NADH-quinone oxidoreductase subunit J	131.50	118.13	−1.11	0.88
METUNv1_460136	METUNv2_290132	NADH-quinone oxidoreductase subunit K	58.88	60.63	1.03	0.91
METUNv1_460137	METUNv2_290133	NADH-quinone oxidoreductase subunit L	637.25	465.50	−1.37	0.83
METUNv1_460138	METUNv2_290134	NADH-quinone oxidoreductase subunit M	372.75	302.75	−1.23	0.77
METUNv1_460139	METUNv2_290135	NADH-ubiquinone oxidoreductase, chain N	523.88	339.07	−1.55	0.64
METUNv1_660132	METUNv2_480138	Ferredoxin-NADP reductase	522.14	879.52	1.68	0.69
METUNv1_590030	METUNv2_420029	Ubiquinol-cytochrome c reductase complex, cytochrome c1	2503.55	1092.90	−2.29	0.58
METUNv1_590031	METUNv2_420030	Ubiquinol-cytochrome c reductase complex, cytochrome b	2945.38	1365.76	−2.16	0.81
METUNv1_590032	METUNv2_420032	Ubiquinol-cytochrome c reductase iron-sulfur subunit (Rieske iron-sulfur protein) (RISP)	3125.54	1240.80	−2.52	0.84
METUNv1_590008	METUNv2_420008	Peroxidase/catalase (Catalase-peroxidase)	3075.82	1883.61	−1.63	0.84
METUNv1_670031	METUNv2_490032	Cytochrome c oxidase subunit 1	5577.29	4801.32	−1.16	0.93
METUNv1_670030	METUNv2_490031	Cytochrome c oxidase subunit 2	4105.55	3835.175	−1.07	0.87
METUNv1_670034	METUNv2_490035	Cytochrome c oxidase subunit 3	2746.54	2227.3	−1.23	0.89
METUNv1_670033	METUNv2_490034	Cytochrome c oxidase assembly protein	1365.325	1525.035	1.12	0.93
METUNv1_580051	METUNv2_410050	Hemin uptake protein hemP (fragment)	1319.89	1517.79	1.15	0.68
METUNv1_580052	METUNv2_410051	Bacterioferritin-associated ferredoxin	930.37	739.50	−1.26	0.80
METUNv1_580053	METUNv2_410052	Bfr, Bacterioferritin	1845.02	2568.53	1.39	0.77

* Each sample represents the average of two biological replicates. Values represent reads per kilobase of coding sequence per million (reads) mapped (RPKM).

### 3.2. Mutagenesis Studies: Methylamine Oxidation

It has previously been shown that deletion of two subunits of NMGS (encoded by *mgsA* and *mgsB*) completely abolished the ability of the strain *M. universalis* FAM5 to utilize methylamine; however the mutants were still able to generate small amounts of *N*-methylglutamate [12]. It has been speculated that the third subunit of the protein (encoded by *mgsC*) can contribute to the residual production. Here we mutated *mgsC* and showed that the lack of the gene eliminates the ability of the strain to utilize methylamine (Table 1).

We also tested involvement of the gamma subunit of the NMGDH (*mgdD*) in methylamine utilization. The *mgdD* is located at the end of the four-gene cluster encoding the NMGDH. The fourth gene has been identified in all microbes possessing the *N*-methylglutamate pathway; however, in contrast to the catalytic subunits (*mgdA* and *mgdC*) and *mgdB*, which are typically quite conserved, the gamma subunit of the enzyme is surprisingly divergent [12]. Relatively low homology (30%–45% amino acid identity) has been observed between the *mgdD* subunit from the *Methyloversatilis* species and the corresponding protein from betaproteobacterial methylotrophs, such as *Methylotenera versatilis* 301 or *Methylophilus methylotrophus* DSM 46235. The gene from *Methyloversatilis* species shared 25% AA identity with the *mgdD* gene from *Agrobacterium tumefaciens*. No homology was observed among *mgdD* genes from betaproteobacteria and gamma subunit of the NMGDH from alphaprotebacterial methylotrophs, such as *Methylocellla silvestris*, *Methylopila* spp, and *Methylobacterium extorquens* spp. The exact function of the *mgdD* gene product is still not known. We mutated the *mgdD* gene in *M.universalis* FAM5, and the mutant strain lost the ability to utilize methylamine (Table 1). These two additional mutations additionally confirmed the importance of the *N*-methylglutamate pathway genes for methylamine utilization by *M. universalis* FAM5.

The transcriptomic study presented above revealed a number of additional functions indicating that the metabolic arrangement of the methylamine utilization in *M. universalis* FAM5 could be more intricate (Figure 1). Two of the most noticeable differences between cells grown on methanol *vs*. grown on methylamine are the overexpression of the heme-containing amine dehydrogenases gene cluster and *fae3* in cells grown on methylamine. We generated a strain of *M. universalis* FAM5 lacking the alpha subunit of the Qhp gene (Δ*qhpA).* The activity of the Qhp enzyme was reduced to background level in the mutant strain (Table 4). However, the strain lacking Qhp gene (Δ*qhpA)* was able to grow on methylamine similarly to wild type (Table 1). The phenotype of the *fae3* mutant is described below.

### 3.3. Mutagenesis Studies: Metabolic Arrangement Downstream from the Methylamine Oxidation

During growth on methanol, formaldehyde, the end product of alcohol oxidation, is oxidized to formate via the H_4_MPT pathway. Formate is either oxidized to CO_2_ to generate energy, or converted to methylene-H_4_F via an FtfL-Fch-MtdA variant of the H_4_F pathway. It has been predicted that the end product of the *N*-methylglutamate pathway is methylene-H_4_F, which could be incorporated directly into the serine cycle. However it is not known how it is oxidized. The FtfL-Fch-MtdA variant of the H_4_F pathway is typically linked to assimilation rather that oxidation [36]. In order to investigate the fate of the methylene-H_4_F in *M. universalis* FAM5 upon growth on methylamine, two possible scenarios were investigated: (1) The H_4_F pathway contributes to methylene-H_4_F oxidation by operating in the reverse direction; (2) The H_4_F pathway in *M. universalis* FAM5 contributes only to formate assimilation, and thus it should not be essential for growth on methylamine. However, in order to generate energy and reducing equivalent from C_1_-oxidation, the *M. universalis* FAM5 cells should possess an additional system for converting methylene-H_4_F to formaldehyde, or transfer C_1_-units from H_4_F to H_4_MPT. One candidate for C_1_-unit decoupling/shuttling is *fae3*, a homolog of formaldehyde activating enzyme, which has a very strong expression during growth on methylamine, but not methanol.

To verify these scenarios, mutations in H_4_F (Δ*mtdA*) and H_4_MPT pathways (Δ*fae1*) were made. In addition we mutated homologs of the formaldehyde dehydrogenase (Δ*fae2* and Δ*fae 3*), and generated multiple mutants (Δ*fae1*Δ*fae2*, Δ*fae1*Δ*fae3*, Δ*fae2*Δ*fae3* and Δ*fae1*Δ*fae2*Δ*fae3*). In addition, a strain lacking *fae1* and *qhpA* was constructed. The mutant phenotypes are shown in Table 1.

Strains lacking *mtdA* or *fae1* genes were not able to grow on methanol or methylamine. Interestingly, revertants were observed for the *fae1*-mutant. The number of revertants arising on methylamine plates was 10 ± 3 per 10^9^ cells plated. The revertant strains grew on methylamine, albeit with a growth defect compared to the wild type strain (the growth rate was about 30% of the wild type). The revertant strains did not restore the ability to utilize methanol for growth. Single *Δfae 2* or *Δfae3* mutations, or the double *Δfae2Δfae3* mutation displayed a growth rate similar to the wild-type strain on both methanol and methylamine. *Δfae1Δfae2* and *Δfae1Δfae3* double mutants were not able to grow on C_1_-compounds, similarly to *Δfae1.* No revertants were observed for *Δfae1Δfae3* mutant upon transfer to methylamine plates. However, the incorporation of the *Δfae3* mutation into the revertant *Δfae1^R^* strain did not abolish the ability of the strain to utilize methylamine. A similar phenotype was observed for a triple *Δfae1^R^Δfae2Δfae3* mutant. The double Δ*fae1*Δ*qhpA* mutant phenotype was similar to the single Δ*fae1* mutant. Like the latter, there were revertants of the double mutant (Δ*fae1*Δ*qhpA^R^*) that regained the ability to utilize methylamine for growth. The frequency of reversion was similar to the single *fae1*-mutant. The triple *Δfae3*Δ*fae1*Δ*qhpA^R^* mutant retained the ability to use methylamine.

**Table 4:** Activity of the heme-containing amine dehydrogenase in wild type and Δ*qhpA-*mutant strains upon growth on methylamine.

Strain	Enzyme Activity (μmol min^−1^ mg^−1^ Protein)
FAM5 (WT)	30 ± 9
Δ*qhpA*	6 ± 3

## 4. Discussion

Methylotrophic capabilities of the members of families *Rhodocyclaceae/Burholderiaceae* have relatively recently been discovered [1,3,4,5,7], and thus not much is known about the pathways for the single carbon utilization in this betaproteobacterial lineage. The *M. universalis* is the first representative of the family isolated in a pure culture and formally described [6]. The genome of the strain has been sequenced opening up new approaches for investigation of pathways for single carbon or methylated multicarbon compounds investigation [23]. Over the past few years the strain became the model system for understanding molecular mechanisms of C_1_-carbon utilization and denitrification [8,12,13].

The mutagenesis performed in this study suggests that the *N*-methylglutamate pathway is the main route for methylamine oxidation. Based on the available genomic and enzymatic data the end product of the N-methylglutamate pathway is methylene-H_4_F [12,16,20]. Thus it could be predicted that, first, the H_4_folate pathway contributes to C_1_-oxidation; and, second, the H_4_MPT-linked transfer should not be required for growth on methylamine. *M. universalis* FAM5 possesses the Mtd-Fch-FtfL variant of the H_4_MPT pathway, which is typically associated with formate assimilation, rather than formaldehyde/methylene-H_4_F oxidation [25,26]. It has been shown that this portion of the pathway is not essential for growth on methylamine in *Methylobacterium spp.* [16,20]. We found here that the *mtdA* mutant is not able to grow on both C_1_-compounds. The phenotypic data indicate that the enzyme of the pathway can play some additional role in C_1_-utilization in *M. universalis*.

We also found that the H_4_MPT pathway is essential for growth on methylamine. That observation indicates that *M. universalis* somehow produces methylene-H_4_MPT during growth on methylamine. Since the heme-dependent amine dehydrogenase (which is predicted to produce formaldehyde) is not essential for growth on methylamine, the C_1_-units entering H_4_MPT pathway should come from methylamine oxidation via the *N*-methylglutamate pathway. Based on the available evidences it could be proposed that the NMGDH releases both methylene-H_4_F and formaldehyde and that the ratio of the produced compounds depends on the intracellular pool of H_4_F. It has been shown that the pool of H_4_F is only a quarter of the pool of H_4_MPT in facultative methylotrophs [37]. In *M.universalis,* the relative expression of the dihydrofolate reductase (*folA*), the only enzyme essential for biosynthesis of H_4_F (*folA*) from a supplied source of folate, showed similar levels of expression in methanol or methylamine grown cells. That suggests that the overall capacity of the cells to produce the folate-cofactor did not change. If methylene-H_4_folate is the only product of methylamine oxidation, the cellular needs for H_4_F should be much higher upon growth on methylamine. Because formaldehyde is a toxic compound, it is reasonable to speculate that formaldehyde is produced only upon the depletion of the H_4_F pool. The Fae1 and/or Fae3 enzymes handle the cellular formaldehyde pool, and deliver C_1_-units into the H_4_MPT pathway for oxidation. However, while this scenario explains the growth deficiencies of the *fae1* mutant, it fails to explain the revertants. It could be speculated that *in vivo* the *N*-methylglutamate dehydrogenase uses both H_4_F and H_4_MPT cofactors. The preferable compound is H_4_F, which could be substituted with H_4_MPT upon depletion of H_4_F and buildup of H_4_MPT (due to the lack of Fae enzymes). The latter scenario is also supported by the previous study of methylamine utilization via the NMG-pathway [16], which showed that the H_4_MPT-biosynthesis, rather than the Fae-driven condensation of formaldehyde, is essential for growth of *Methylobacterium extorquens* PA1 on methylamine.

The initial reconstruction of the methylotrophy in methylotrophic *Rhodocyclaceae/Burholderiaceae* has been made based on enzymatic and genetic investigations [13,38,39]. In this study we further evaluated pathways for aerobic utilization of methanol and methylamine via transcriptomics and mutagenesis. The updated reconstruction of the core metabolic pathways in *M. universalis* FAM5 is shown in Figure 1. It has been demonstrated that methanol oxidation via PQQ-dependent enzymes is operating as a *redox arm* and is coupled with ATP generation with 0.6–1 mol of ATP produced per 1 mol of methanol oxidized [40]. Thus, the growth on the C_1_-alcohol is predicted to be reducing-power limited. Interestingly, the transcriptomic studies show that cells of *M. universalis* FAM5 expressed complex III (cytochrome *bc*_1_). As the majority of the cellular energy needs are fulfilled by the first step of methane oxidation, it could be speculated that *M.universalis* FAM5 uses complex III to supply reducing power for carbon assimilation upon growth on methanol. Methylamine oxidation in *M.universalis* FAM5 might also be connected to NADPH production, via a ferredoxin-NADP reductase. An uphill electron transfer has been proposed for a number of chemolitotrophs [41,42,43], but it has never been investigated in non-phototrophic methylotrophs. Here we present the first evidence that methylotrophic bacteria might develop a mechanism for restoring reducing power upon growth on C_1_-compounds. The exact role of the complex III and FNR in cellular energetics will be evaluated in further investigations.

## 5. Conclusions

Overall, the metabolic arrangement of C_1_-utilization in *M. universalis* appears to be quite complex and to some degree redundant. Methylamine utilization is supported by two different enzymatic systems: the *N*-methylglutamate pathways and heme-dependent amine dehydrogenase. Genes encoding both enzymatic systems are chromosomally co-located and show a very high level of expression upon switch from growth on methanol to growth on methylamine. Like other heme-containing amine dehydrogenases, the enzyme from *M. universalis* FAM5 is capable of direct conversion of methylamine to formaldehyde. However, from the Δ*qhpA* phenotypic data it could be suggested that the enzyme is not essential, at least under the growth condition tested, for methylamine utilization.

The functional implication of the enzymatic redundancy of the C_1_-utilization is not apparent in the experiments described here. It could be speculated, that in the presence of heterogeneous substrates, the simultaneous activation of different enzymatic pathways might provide some benefit. However, it remains to be determined via additional simulation experiments or *in-situ* investigations.

## Supplementary Files

Supplementary File 1
