# Supplementary material for: C1-Pathways in Methyloversatilis universalis FAM5: Genome Wide Gene Expression and Mutagenesis Studies
Source: Microorganisms. 2015 Apr 9;3(2):175–97. doi: 10.3390/microorganisms3020175 (PMC5023235; doi:10.3390/microorganisms3020175)
Supplement: Supplementary File 1 [file microorganisms-03-00175-s001.docx]

**Supplementary Information**

**Table S1.** Primers used in this study.

| **Gene/Locus Tag** | **Primer Sequence** | **Primer Name** |
| --- | --- | --- |
| *fae 3/*METUNv2_520523 |  |  |
| upstream flank | GAGGACGTCGGTTTGACCAAGGCGGACAGG | Fae3UP/F-AatII |
|  | GAGGGTACCGCACCACCTGTGCGCGGACTCATGTG | Fae3UP/R2-KpnI |
| downstream flank | GAGCCGCGGCGCCGCAGCCACGTCGCTGCAG | Fae3DW/F-SacII |
|  | GAGGAGCTCGACGCCGACGCCCGCACCCAGCAGCG | Fae3DW/R-SacI |
| *fae 2/*METUNv2_480039 |  |  |
| upstream flank | GAGGACGTCGCGCTGCCGAGGTGATGGTGG | Fae2UP/F-AatII |
|  | GAGGGTACCGTTGGCGTACGCGGGAGGCGCGC | Fae2UP/R-KpnI |
| downstream flank | GAGGGGCCCAGGTCGGTCAGCAGCACGG | Fae2DW/F-ApaI |
|  | GGGAGCTCGGTTCTGGCTTTCGTTCAGGATG | Fae2DW/R-SacI |
| *fae1/*METUNv2_410093 |  |  |
| upstream flank | GAGGACGTCGCTCGACATCGAAGCGCTGAAG | Fae1UP/F-AatII |
|  | GAGGGTACCTCATTTACAAGGCAGTTATAGG | Fae1UP/R-KpnI |
| downstream flank | GAGCCGCGGCGACTCGGTTACAGCACACGTG | Fae1DW/F-SacII |
|  | GAGGAGCTCGCGGTGATCCAGATACCGATCGC | Fae1DW/R-sacI |
| *mtdA/*METUNv2_290311 |  |  |
| upstream flank | gacagatctGGTGAAGGCGTGCTCGG | MtdA-upF/BglII |
|  | gacggtaccTGTGATGTCTCGGATGTCTGG | MtdA-upR/KpnI |
| downstream flank | gacgggcccGCGATGAGCCAGATCAAGG | MtdA-dwF/ApaI |
|  | gatgagctcGCGCGAATGGCTGAACCGGGTG | MtdA-dwR/SacI |
| *mch/*METUNv2_410090 |  |  |
| upstream flank | GGGACGTCGACATTACGGCCAGCGATGC | mch-UP/F-AatII |
|  | GAGGTACCGGCTGTGCGCCGGTCAGTAC | mch-UP/R-KpnI |
| downstream flank | GACCGCGGAGCTGCTGGCGAAGTCCTTC | mch-DW/F-SacII |
|  | GAGAGCTCGCTGCATCAGCGCATCGTC | mch-DW/R-SacI |
| *mdsC/*METUNv2_580117 |  |  |
| upstream flank | GGAGACGTCGCACAAGGGTCTTGAGGAAGG | NmgsCup-AatII/F |
|  | GAGGGTACCGCGCGGGAGGGCGGATCG | NmgsCup-Kpn/R |
| downstream flank | GAGCCGCGGAACATGGAACTGACCACG | NmgsCdw-SacII/F |
|  | GGGAGCTCGAGGTGACGGCGGGTCAGAC | NmgsCdw-SacI/R |
| *mgdD/*METUNv2_580113 |  |  |
| upstream flank | GAGGACGTCGGCGAATGGGGCTG | Nmgdh4up-AatII/F |
|  | GAGCCATGGCTTCGACCTGGGTCCAC | Nmgdh4up-Nco/R |
| downstream flank | GAGCCGCGGCAATAACAAACAGCGCG | Nmgdh4dw-SacII/F |
|  | GAGAGCTCGGACAGACGCTCGAGGAACACG | Nmgdh4dw-SacI/R |

**Table S1.** *Cont*.

| *qhpA/*METUNv2_580127 |  |  |
| --- | --- | --- |
| upstream flank | atgaattctgcaaggacaccggcgacagcaa | Up flank F |
|  | atggtaccaatccatcatcaacgcctcgctga | Up flank R |
| downstream flank | tagttaactcttcggtcaccacgcagcacagc | Down flank F |
|  | tagagctcgacaggacgcgcacttggtattg | Down flank R |
| *qhpX/*METUNv2_580128 |  |  |
| upstream flank | atgaattctcttcggtcaccacgcagcacagc | Up flank F |
|  | tacttaacccgacgccaatctgctctatgtg | Up flank R |
| downstream flank | atggtaccgacaggacgcgcacttggtattg | Down flank F |
|  | tagagctcagttcgtcaccgaccaggttgaag | Down flank R |
